# Questionnaire-based survey on distribution and clinical incidence of canine babesiosis in France

**DOI:** 10.1186/1746-6148-9-41

**Published:** 2013-02-28

**Authors:** Lénaïg Halos, Isabelle Lebert, Isabelle Chao, Gwenaël Vourc’h, Christian Ducrot, David Abrial, Jean-François Ravier, Jacques Guillot

**Affiliations:** 1 Merial, 29 avenue Tony Garnier, 69007, Lyon, France; 2 INRA, UR346, F-63122, Saint-Genès Champanelle, France; 3 BIPAR, UMR ENVA, ANSES, UPEC, Ecole Nationale Vétérinaire d’Alfort, UPE, 94704, Maisons-Alfort, France

**Keywords:** *Babesia canis*, Canine babesiosis, Incidence rates, Geographic distribution, France

## Abstract

**Background:**

The causative agent of canine babesiosis is the protozoan *Babesia canis*, transmitted by the tick *Dermacentor reticulatus* within France. While the parasite can be found everywhere in France however cases of infection are associated with distinct geographical foci. The aim of the study was to evaluate the clinical occurrence of canine babesiosis diagnosed in veterinary clinics in order to propose an updated map of the disease distribution in France.

**Results:**

Questionnaires were sent via email to all canine veterinary clinics in continental France. Information collected included the number of babesiosis cases diagnosed in 2010, the number of veterinary practitioners and the location of the clinic. The total number of dogs and practitioners per administrative department were used to define the reference population. The annual incidence rate of canine babesiosis per department was calculated as the ratio between the number of babesiosis cases reported by the clinics and the total number of dogs in the clinics of the same department. Data were geo-referenced for map construction (Quantum GIS version 1.7.4). The overall annual incidence rate of clinical babesiosis among the surveyed population was 1.07% (CI95 1.05-1.09) with geographical variations between departments, ranging from 0.01% to 16.05%. Four enzootic areas were identified: South-West, Center, East and Paris area. The South-West region should be considered as a hyper-enzootic area with the higher incidence rates.

**Conclusion:**

Our results confirmed the burden of canine babesiosis in France. In the context of tick-borne disease emergence in Europe, the risk for canine babesiosis may become more significant in other European countries in the coming years.

## Background

Babesiosis is an emerging tick-borne hemoprotozoosis affecting many mammalian species worldwide. Pathogenesis is due to intra-erythrocytic multiplication of apicomplexan parasites of the genus *Babesia,* which are amongst the most ubiquitous and widespread blood parasites. Consequently, Babesiosis has a considerable global economic, human health, and veterinary impact [1]. *Babesia sp.* are closely related to another apicomplexan protozoan, *Plasmodium sp.*, the causative agent of malaria. The phylogenetic proximity and numerous similar biological features [2] between *Babesia* and *Plasmodium* have earned animal babesiosis the moniker of “animal malaria” [1,3,4]. The different species of *Babesia* are transmitted by hard ticks and are capable of infecting a wide variety of vertebrate hosts with a high host specificity. Each specific host is competent in maintaining the transmission cycle of a given species of *Babesia*[1].

In France, two species of *Babesia* are considered to infect dogs. The most prevalent, *Babesia canis,* is transmitted by the tick *Dermacentor reticulatus,* also called the European dog tick or ornate dog tick. The second, *B. vogeli* is transmitted by the brown dog tick, *Rhipicephalus sanguineus,* and is restricted to the south of the country [5]. Both species are morphologically similar and classified as large *Babesia* (i.e. larger than the radius of a red blood cell) [6,7]. The species *B. vogeli* is generally thought to be less pathogenic, causing subclinical, mild or moderate symptoms in adult dogs [6,8,9] whereas *B. canis* remains a life-threatening parasite.

Dogs infected by *B. canis* typically present with the acute form of babesiosis, which is characterized by the association of both febrile and hemolytic syndromes. General findings such as weakness, mucous membrane pallor, depression, lymphadenopathy, splenomegaly, and general malaise are common. Laboratory studies may document anemia, thrombocytopenia, hypoalbuminemia, and bilirubinuria [6].

Microscopy remains the simplest and most accessible diagnostic test for most veterinarians and during acute infections is reasonably sensitive for detecting intra-erythrocytic parasites in Giemsa or Wright’s stained blood smears from capillary beds [6,8]. Babesiosis rarely resolves spontaneously and requires specific treatment such as imidocarb dipropionate, which is the reference drug for the treatment of animal babesioses in France [5,6].

France is known to have a high enzootic prevalence of babesiosis with a heterogeneous distribution [5,6]. The disease is essentially observed in two regions: a large region in the southwest/west extending from Languedoc to Sologne, and a region centred in Lyon and spreading to the southwest of Burgundy and the centre of France [6]. Very few large scale surveys have been conducted in France to assess the impact of the disease in the general dog population. Moreover, data on clinical incidence are scarce as access to unpublished field-observed clinical cases is limited. Most studies were conducted in restricted areas or did not take into account the exposed population [6,10]. A better overview of the disease epidemiology is a key factor for improvement of control strategies [11].

The present study was based on a questionnaire survey sent via email to 4400 canine veterinary clinics in continental France asking them to report the number of babesiosis diagnosed in 2010. The dog population per veterinary surgeon was estimated according to reference figures (i.e. number of companion animal veterinarians and number of dogs per administrative department in France) and the annual incidence rate of canine babesiosis was calculated per geographic area.

## Methods

### Definition of cases and questionnaire

The cases were defined as clinical cases of babesiosis of dogs diagnosed by veterinarian practitioners in their practices over the course of a year (2010).

Standardised questionnaires were sent by email in June 2011 to canine clinics from continental France (4400 clinics corresponding to 10,652 canine veterinarian practitioners in 96 administrative departments). The total number of questions was limited in order to encourage participation: the zip code of the clinic, the number of cases diagnosed in the clinic for the period of 2010 and the number of veterinarians treating companion animals at the clinic. The survey was anonymized and veterinary practitioners participated on a voluntary basis. No approval by an ethics committee was required for the applied methodology.

### Estimate of the dog population

In order to calculate the percentage of the dog population that contracted babesiosis during the period of the study, reference data on the veterinarian and dog populations were required. As it was not possible to obtain the annual number of dogs that presented to each clinic, the estimated dog population per companion animal veterinarian in each administrative unit was calculated as the total number of dogs in the administrative unit divided by the number of companion animal veterinarians in the unit.

The yearly updated number of companion animal veterinarians per administrative department was kindly provided by the French Veterinary Syndicate (SNVEL, Syndicat National des Vétérinaires d’Exercice Libéral, Paris). This database comprises information on veterinarians whose practice is totally devoted to companion animals as well as veterinarians whose practice is shared between companion and production animals, therefore the number of companion animal practitioners calculated for this study was the sum of the total number of companion animal veterinarians and half the number of veterinarians of mixed practice.

To date, there is no official figure available for the canine population in European countries. The most accurate estimates are those obtained from pet food producers. The estimated number of dogs per administrative department in France was kindly provided by the French Syndicate of Pet food producers (FACCO, Chambre Syndicale des Fabricants d’Aliments pour Chiens, Chats, Oiseaux et autres animaux familiers, Paris). According to this database, the overall dog population is estimated to be approximately 8,013,700 in continental France.

The mean number of dogs per companion animal veterinarian in each administrative department (data not shown) was calculated as follows:

$$\mathrm{meanDOG}{}_{\mathrm{dep}}=\;\mathrm{DOG}{}_{\mathrm{dep}}\;/\;\left(\mathrm{VET}{}_{\mathrm{companion}}\;+\;0.5\;\times \;\mathrm{VET}{}_{\mathrm{mixed}}\right)$$

where DOG_dep_ is the estimated number of dogs per administrative department, VET_companion_ is the number of companion animal veterinarians per administrative department and VET_mixed_ is the number of veterinarians of mixed practice per administrative department.

### Computation of the annual incidence rate

The number of canine babesiosis cases, as well as the number of companion veterinarians who participated in the study per department was obtained by grouping returned questionnaires from the same zip code.

Annual canine babesiosis (CB) incidence was defined as the number of new cases occurring over the one year period in the area of interest (each department and the whole territory). The annual canine babesiosis incidence rate (CB Inc_dep_) was calculated for each area as the ratio of the total number of affected dogs in all the clinics of the area that answered the questionnaire to the estimated dog population in these clinics following the formula:

$$\mathrm{CB}\;\mathrm{Inc}{}_{\mathrm{dep}}=\;\sum_{\mathrm{dep}}\mathrm{CB}\;\times \;100/\left(\left(\sum_{\mathrm{dep}}\mathrm{CAV}\right)\times \;\mathrm{meanDO}G_{\mathrm{dep}}\right),$$

where CB corresponds to the cases of canine babesiosis registered during the study, CAV corresponds to the companion animal participating veterinarians and *meanDOG*_*dep*_ corresponds to the mean number of dogs per companion animal veterinarian per department as described above.

The annual incidence rate was computed at department and national levels. A score test was performed to compare the annual incidence rate of each department with the national incidence rate considered as the reference [12]. No score test was performed when the number of babesiosis cases was less than five. Departments were categorised into three classes according to the difference from the reference: no difference, significantly lower and significantly higher than the reference.

To check that the numbers of clinical cases did not depend upon the response rate, we draw the distribution of the response rate as a function of the annual incidence rate.

### Map building

The geo-referencing of the French departments was kindly provided by the French National Mapping Agency (IGN, Saint Mandé, France). The incidence rate per department was spatially referenced to department numbers and mapped using Quantum-GIS Software (version 1.7.4). All departments with an annual incidence rate significantly different from the annual incidence rate at the national level were mentioned on the map (score test).

## Results

### Questionnaire response rate and raw data collection

Responses were received from 748 clinics in continental France (representing 1,496 veterinarians) covering the entire country except for two out of 96 departments (Territoire-de-Belfort and Corse-du-Sud) from which no answer was received. Between 1.8% (department 23, Creuse) and 39.6% (department 58, Nièvre) of the veterinarians from each department answered the questionnaire. The average response rate was 14.8%.

**Figure 1:**
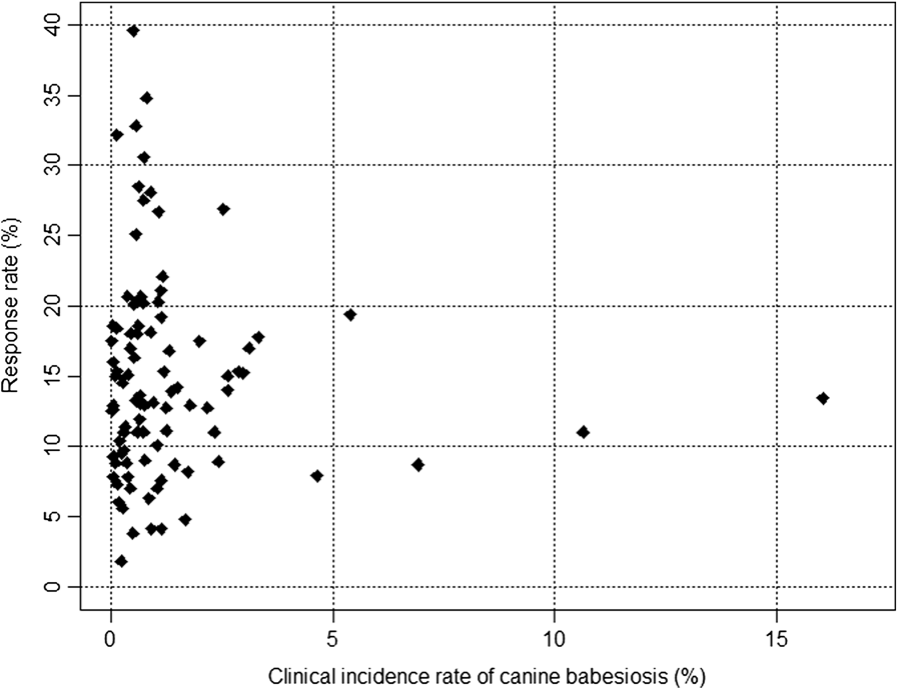
Distribution of the departments according to the incidence rate of canine babesiosis and the response rate of the veterinarians to the survey in continental France.

The total number of babesiosis cases registered in 2010 by the veterinarians that answered the questionnaire was 12,064. Among the 748 clinics, 149 reported no case of babesiosis, while the 599 remaining reported between one to 320 cases. In all departments, at least one case was reported. No correlative pattern was observed between the response rate and the number of clinical cases reported (Figure 1).

### National annual incidence rate

The overall annual incidence rate of clinical babesiosis amongst the French dog population was 1.07% (95% confidence interval (CI95) 1.05-1.09) with geographical variations among the departments (from 0.01% to 16.05%). The majority of the departments (n = 58 on 93) had an annual incidence rate below 1.0%; 16 departments presented an incidence rate between 1.0% and 1.5%, 15 between 1.5% and 5.0%, and 4 had a rate above 5.0% (with two departments reaching values of 10.64% (CI95 9.62– 11.66) and 16.05% (CI95 14.79-17.32). In the department Haute-Corse, one case was reported but the incidence rate could not be calculated because the total number of dogs in this department was not available.

The three departments with the highest incidence rates (Ariège-16.05%, Gers-10.64% and Jura-6.93%) had veterinarian participation rates of 13.4%, 11.0% and 8.7%, respectively. For these three departments, the number of babesiosis cases reported by a veterinary practice ranged from 15 to 320. The three departments that had the lowest incidence rates (Alpes-Maritimes 0.01%, Côtes-d'Armor 0.03% and Var-0.04%) had participation rates of 17.5%, 12.5% and 18.6%, respectively. For these three departments, the number of babesiosis cases reported by a veterinary practice ranged from 0 to 3.

### Geographic distribution

**Figure 2:**
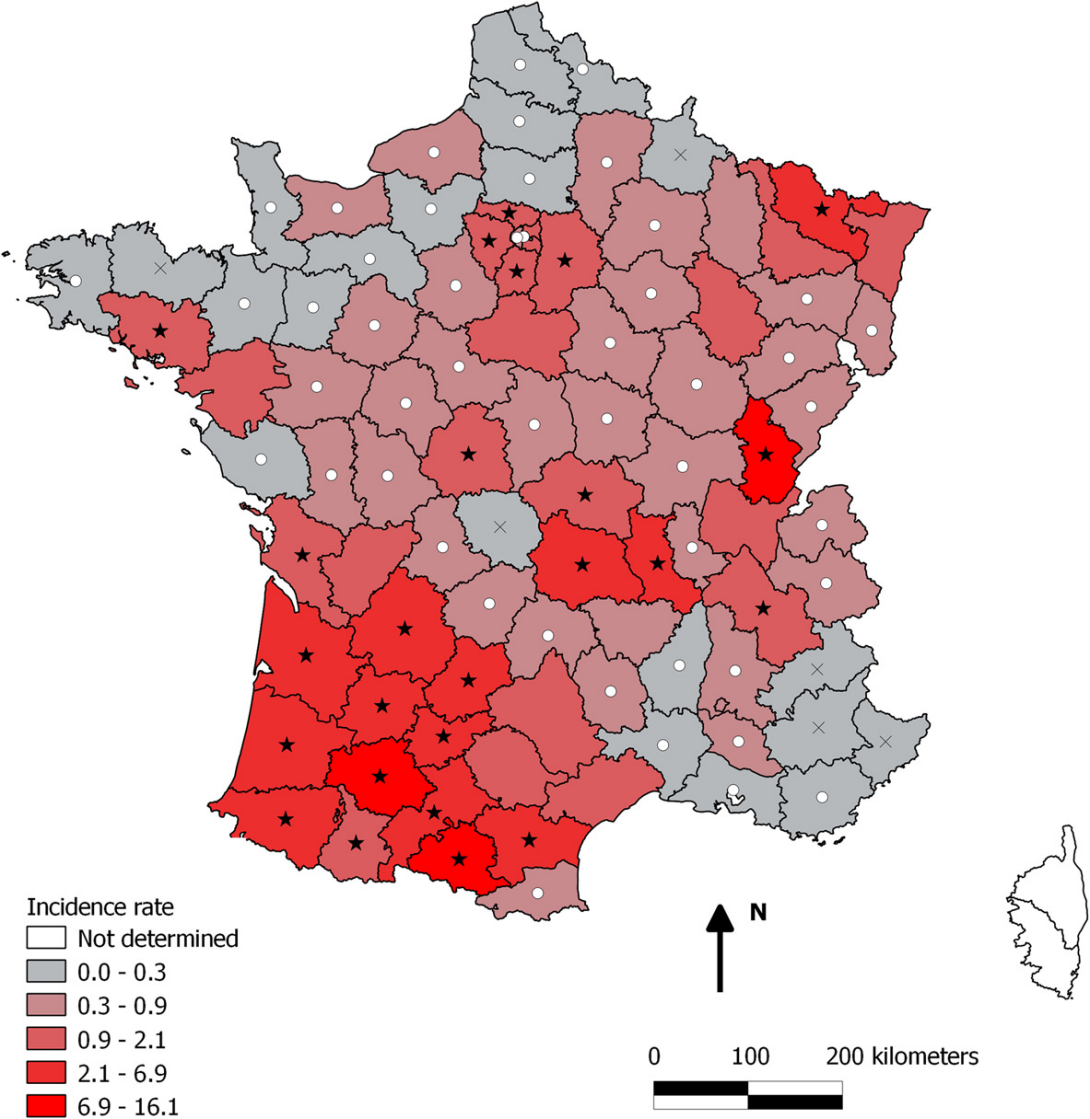
**Geographical distribution of the estimated incidence rate of canine babesiosis among general dog population in French departments in 2010.** The color gradient (from grey to red) indicates an increasing incidence rate. Departmental incidence rate was significantly lower than the national incidence rate (○) or significantly higher than the national incidence rate (★).

Babesiosis was observed on the whole territory and displayed a highly variable pattern (Figure 2). The distribution map allowed identification of four major enzootic areas:

– south-west France (departments: Ariège, Gers, Landes, Aude, Haute-Garonne, Pyrénées-Atlantiques, Tarn-et-Garonne, Lot, Gironde, Dordogne, Lot-et-Garonne, Charente-Maritime, Hautes-Pyrénées) showed particularly high incidence rates. Notably, two departments Ariège and Gers reported a very high clinical incidence of 16.1% and 10.6%, respectively.

– the Auvergne region in the centre of France (departments: Loire, Puy-de-Dôme, Allier),

– the suburb of Paris (departments: Essonne, Seine-et-Marne, Val-d’Oise);

– one region in the east of France (department of Moselle).

In 14 departments, the annual incidence rate was not significantly different from the national annual incidence rate (*p* > 0.05). Forty-eight departments had an annual incidence rate significantly lower than ‘the national average’, while 25 departments had an annual incidence rate significantly higher than ‘the national average’ (*p* < 0.05, Figure 2). For six departments, the number of related babesiosis cases was not sufficient (less than five cases per department) to apply the score test.

## Discussion

Practitioner questionnaire surveys provide a highly-informative approach for estimating the incidence of animal diseases and contribute to a better understanding of the evolution of the diseases over large geographic areas. The major biases of questionnaire-based epidemiological surveys are the potential inaccuracies linked to failures in diagnosis (i.e. over or under estimation), limited response rates or reliability of the reference population estimates [13,14]. These biases were considered in the present study and efforts were made to reduce any potential impact. The reference population figures were defined with the most accurate canine population or veterinary distribution databases available in order to provide a reliable analysis. The mean response rate of 14.8% was associated with a widespread coverage of the country and thus facilitated the construction of a very accurate model. Under-estimation of infection rates could result from cases that were not presented to the veterinary surgeon or from diagnostic failure. The latter cases may principally occur in the case of chronically infected dogs or atypical presentations. Over-estimation might be due to false diagnosis by the veterinarian practitioner. Differential diagnoses may include a number of causes of anaemia, such as other vector-borne diseases [5,8,15]. Generally, babesiosis due to *Babesia canis* presents with acute symptoms [8], however response to imidocarb is a good indicator for a field-diagnosis of babesiosis and diagnosis conducted on the basis of clinicopathological findings have a high accuracy rate (93.5%) [15], making it possible to rely on the collected data. A possible bias associated with a variable response rate is that the veterinarian practitioners who answered the questionnaire may have been those who more frequently observe clinical babesiosis in their practice and therefore feel more implicated in the epidemiological investigation of the disease. If so, this may have resulted in an overestimation of the incidence rates in areas with low response rates. However, the impact of this potential bias seems limited as there is no correlation between the incidence rates and the response rate Figure 1 (i.e. high incidence rates are not reported in departments where the response rate was low and conversely). This along with the wide coverage of the country, ensured that any spatial differences observed in the incidence rates between departments highlighted the spatial distribution of the disease rather than being randomly associated with variations in response rates.

The present study is, to our knowledge, the first study offering an evaluation of the incidence of babesiosis among the at-risk dog population in France. The study confirms the broad distribution of the disease over the country and a very high degree of heterogeneity of distribution. In addition, unexpectedly high figures of the annual incidence rates of canine babesiosis in France were observed. Mean annual incidence observed across the country was 1.07%, with values ranging between 0.01% and up to 16.05% according to the geographic localization. The higher values concerned only a few French departments. In those hyper-enzootic areas, up to 16% of the entire dog population was diagnosed with clinical babesiosis during 2010.

To understand the impact of this level of incidence, it is interesting to compare it with World Health Organization (WHO) classification for malaria distribution. WHO considers areas where annual incidence of malaria is higher than 10% as hyper-endemic areas and areas where the annual incidence is above 0.1% as high-transmission areas for malaria [16]. According to this classification, if applied to canine babesiosis, France can globally be considered as a high transmission area of *Babesia canis*.

Observed distribution and incidence rates corroborated data from previous studies conducted in France [6,10]. Southwest France remains identified as a very important enzootic area and three other regions recognized as highly enzootic have been previously identified as endemic for canine babesiosis. The central region described previously as centred around Lyon [6] appears to be located more westerly and covers the Massif Central area. Additional observations, such as the identification of four core areas for the disease had never previously been outlined. Further studies on a smaller scale should be conducted in those areas in order to investigate the factors influencing the local expression of the disease. Babesiosis is a disease known to be highly dependent on specific biotopes with a very heterogeneous distribution on a very small scale [6]. Those factors are probably correlated with a favourable environment for the *D. reticulatu*s tick. *D. reticulatus,* is a three-stage polytropic, and hydrophilic tick. Larvae and nymphs infest micro-mammals and are endophilic, living in their hosts burrows, whereas adults are exophilic with a tropism primarily for dogs and at a lower extent to ungulates such as horses, sheep and cattle. This tick is adapted to temperate climate with a strong preference for open areas with a high humidity level, especially riverbanks, paths sides, parkland and wasteland [17]. The hypothesis of the strong correlation between *D. reticulatus* and canine babesiosis distribution is supported by observations from several studies [18,19].

The occurrence of *B. vogeli* is scarce in France but should be considered in areas with *R. sanguineus*, the vector [5], where it may contribute to increasing the prevalence of the disease when the two tick species co-exist such as in southwest France.

## Conclusions

Our results rank babesiosis among the main canine infectious diseases in France and highlight the clinical significance of the disease. In the context of tick-borne disease emergence in Europe [11], the risk for canine babesiosis may become more significant in other European countries. The distribution of the disease in Europe is known to be expanding but currently not well described. Similar studies conducted at the European level would offer an improved knowledge on the reality of the disease in the field and enable adapted monitoring.

## Competing interests

The authors declare that they have no competing interests.

## Authors’ contributions

LH conceived the study and drafted the manuscript. IC handled the questionnaire emailing, the contact with vet clinics and collected data on vet and dog population for reference figures. IL carried out the geospatial and statistical analysis and participated in the draft of the manuscript. GV participated in the design of the study and helped to draft the manuscript. CD participated in the design of the study and its coordination and helped to draft the manuscript. DA participated in the geospatial and statistical analysis conception. JFR was implicated in the collection of vet addresses, questionnaires and in the control of the accuracy of the answers. JG participated in the conception of the study design and helped to draft the manuscript. All authors read and approved the final manuscript.
